# Trends and Drivers of Unmet Need for Family Planning in Currently Married Tanzanian Women between 1999 and 2016

**DOI:** 10.3390/ijerph20032262

**Published:** 2023-01-27

**Authors:** Abdon Gregory Rwabilimbo, Kedir Y. Ahmed, Jackline Boniphace Mshokela, Amit Arora, Felix Akpojene Ogbo, on behalf of the Global Maternal and Child Health Research Collaboration (GloMACH)

**Affiliations:** 1 Ifakara Health Institute, Dar Es Salaam 14112, Tanzania; 2 Medical Team International, 4th Floor Plot No.96. Mikocheni Light Industrial Area, New Bagamoyo Road, Dar Es Salaam 14112, Tanzania; 3 International Rescue Committee, Kasulu 47301, Tanzania; 4 Rural Health Research Institute, Charles Sturt University, Orange, NSW 2800, Australia; 5 Translational Health Research Institute, Western Sydney University, Campbelltown, NSW 1797, Australia; 6 School of Health Sciences, Western Sydney University, Campbelltown Campus, Locked Bag 1797, Penrith, NSW 2751, Australia; 7 Discipline of Child and Adolescent Health, Sydney Medical School, The University of Sydney, Westmead, NSW 2145, Australia; 8 Health Equity Laboratory, Campbelltown, NSW 2560, Australia; 9 Oral Health Services, Sydney Local Health District and Sydney Dental Hospital, NSW Health, Surry Hills, NSW 2010, Australia; 10 Riverland Academy of Clinical Excellence (RACE), Riverland Mallee Coorong Local Health Network, SA Health, Government of South Australia, Berri, SA 5343, Australia

**Keywords:** trends, drivers, unmet need, family planning, married, women, Tanzania, demographics and health survey

## Abstract

The current study investigated the trends and factors associated with the unmet need for family planning (FP) for limiting and spacing births among married Tanzanian women between 1999 and 2016. The study used Tanzania Demographic and Health Survey (TDHS) data for the years 1999 (*N* = 2653), 2004–2005 (*N* = 2950), 2010 (*N* = 6412), and 2015–2016 (*N* = 8210). Trends in the unmet need for FP were estimated over the study period. Multivariable multinomial logistic regression models were used to investigate the association between community-level, predisposing, enabling, and need factors with the unmet need for FP in Tanzania. The results showed no significant change in percentage of married women with an unmet need for birth spacing between 1999 and 2016. The proportion of married women with an unmet need for limiting births decreased from 9.5% (95% confidence interval (CI): 7.9%, 10.6%) in 1999 to 6.6% (95% CI: 5.9%, 7.3%) in 2016. Residing in a rural area, parity between 1–4 and 5+, visiting a health facility for any health services within twelve months, and planning to have more children (after two years and/or undecided) were factors positively associated with the unmet need for FP-spacing. Women with parity of 5+ were more likely to experience an unmet need for FP-limiting. Women’s age between 25–34 and 35–49 years, women’s employment status, watching television, women’s autonomy of not being involved in household decisions, and planning to have more children were factors associated with lower odds of having an unmet need for FP-spacing. Women’s age between 25–34 years, watching television, autonomy, and planning to have more children were factors with lower odds of having an unmet need for FP-limiting. Improving FP uptake among married Tanzanian women can reduce the unmet need for FP. Therefore, reducing unmet needs for FP is attainable if government policies and interventions can target women residing in rural areas and other modifiable risk factors, such as parity, health facility visits, planning to having more children, employment, watching television, and women’s autonomy.

## 1. Introduction

Adequate access to modern family planning (FP) methods allows people to make informed choices about their sexual and reproductive health and enables them to attain the desired number of children and birth spacing [1]. In contrast, the unmet need for FP increases the risk of unintended pregnancy and its complications, including unsafe abortion, maternal and infant deaths, and has the potential to limit the development of a community [1]. The World Health Organization (WHO) described the unmet need for FP as women who are fecund and sexually active but are not using any method of contraception and report not wanting any more children or wanting to delay the birth of the next child [2]. According to the Demographic and Health Survey (DHS) report, the unmet need for FP (limiting births) among currently married women includes those who are not using contraceptive, not pregnant or postpartum amenorrheic, and fecund who do not want more children, while the unmet need for spacing births among women includes those who are married but are not using contraceptives, not pregnant or postpartum amenorrheic, and fecund who want the next child in 2 years or more, want a child but undecided about timing, or undecided when to have a child [3].

Evidence shows that the use of FP methods provides many health and socio-economic benefits to the mother, child, family, and community [1]. For the mother, FP use reduces the risk of unsafe abortion, the number of unintended pregnancies and associated complications, and improves women’s health by allowing mothers to have enough time to recover from birth-related health issues. For the child, FP reduces the risk of stillbirth and infant mortality by preventing closely spaced and unintended births, but it also allows the baby to have more time for breastfeeding [1]. For the family and community, FP use can accelerate the socioeconomic development of the household and community by having more women in the workforce [4].

In the past three decades, global health organizations have made efforts to improve maternal health and wellbeing. The Safe Motherhood Initiative (convened in Nairobi in 1987 by the World Bank, WHO, and United Nations Fund for Population Activities [UNFPA]) recognized FP as one of the core strategies to reduce maternal morbidity and mortality as well as improve child survival [2,4]. In many parts of the world, access to and use of modern FP methods have increased over time, but improving availability and accessibility to modern FP methods in Sub-Saharan African countries (including Tanzania) have stalled [5]. In these settings, an estimated one in four women of reproductive age who want to avoid pregnancy do not have access to (and therefore do not use) any modern FP methods [6,7].

According to the 2015–2016 Tanzania Demographic and Health Survey (TDHS) report [8], the national unmet need for contraceptives was higher (22%) in Tanzania than the global average of 12% [9], with considerable variations across subnational regions in the country [8,10,11]. Previous studies using data from low–and middle-income countries (LMICs) [12,13] have reported that residing in poor households, low level of mother’s and father’s education, traveling a long distance to reach a health facility, receiving health professional advice, media exposure, low parity, and middle-aged women (25–34 years) were factors associated with the unmet need for FP. In Tanzania, past studies on the unmet need for FP had limitations: (i) a lack of information on trends in the unmet need for FP methods; and (ii) no studies differentially examined the drivers and barriers of the unmet need for FP (limiting and spacing births).

Comprehensive insight into the unmet need for contraceptives among women of reproductive age is essential for policy makers and health professionals to address Tanzania’s progress towards the country’s commitment to the FP 2030 initiative (i.e., increase Tanzania’s modern FP for all women to 42% by 2025) [1,14]. The evidence would also be useful in tracking the country’s progress towards the Sustainable Development Goals (SDGs-3.7, ensuring universal access to sexual and reproductive health care services, including FP, information, and education) [15]. Additionally, the study findings may have relevant implications for other LMICs with similar demographic, health, and population structures. Thus, this study investigated the trends and factors associated with the unmet need for FP (for limiting and spacing births) among currently married women in Tanzania from 1999 to 2016.

## 2. Materials and Methods

### 2.1. Data Sources

The study used TDHS data from 1999 to 2016, 1999 (*N* = 2653), 2004/05 (*N* = 6950), 2010 (*N* = 6412), and 2015–16 (*N* = 8210). The National Bureau of Statistics (NBS), Office of the Chief Government Statistician (OCGS) in Zanzibar, and Inner-City Funds collected the data. The project was funded by the Government of United Republic of Tanzania, Global Affairs Canada, and the United States Agency for International Development (USAID) [16]. Data for maternal health, including FP, child health, infant nutrition, and other health-related data, were collected based on a nationally representative population in Tanzania [17,18,19,20].

The data were collected from eligible women aged 15–49 years who were married or cohabiting and were residents in the household 24 h prior to the survey using a two-stage stratified cluster sampling technique. In stage one, enumeration areas (EAs) were selected proportional to each geographical zone of Tanzania. The EAs were based on the 1988, 2002, and 2012 Tanzania Population and Housing Censuses, respectively [21,22,23]. In stage two, a systematic random sampling technique was used to select households after the complete household listing was conducted in each EAs. The response rates in the surveys were 98% in 1999, 97% in 2005–2004, 96% in 2010, and 97% in 2015–2016. The full methodological approaches used in the surveys are provided in the respective TDHS reports [17,18,19,20].

The present study was conducted based on a total weighted sample of 24,225 married and/or cohabiting women who were not using FP on the day of the interview but reported a willingness to use modern FP methods [24,25,26,27].

### 2.2. Study Setting

The United Republic of Tanzania has 31 regions (28 in mainland Tanzania and 3 in Tanzania Island) with a total of 184 districts. Geographically, Tanzania is divided into regions, districts, divisions, wards, and villages. Tanzania is the largest country in East Africa, covering a total of 947,300 km^2^. Based on 2022 National Census results, Tanzania has a total of 61,741,120 people, of whom 30,053,130 (48.8%) are men and 31,687,990 (51.3%) are women [28]. The 2015–2016 TDHS indicated that the total fertility rate was 5.2 [19]

### 2.3. Outcome Variable

The study outcome was the unmet need for FP, measured as the proportion of women who: (1) are not pregnant or postpartum amenorrhoeic and are considered fecund and want to postpone their next birth for 2 or more years or stop childbearing altogether, but are not using a contraceptive method; (2) have a mistimed or unwanted current pregnancy; or (3) are postpartum amenorrhoeic and their last birth in the last 2 years was mistimed or unwanted [10]. The unmet need for FP was divided into: (i) unmet need for spacing births; and (ii) unmet need for limiting births (that is, women who want to space or limit births but are not currently using FP methods) [10]. Dividing the unmet need for FP into the need for spacing and limiting births provides additional and detailed information about issues related to non-use of FP methods among currently married women in Tanzania.

In this study, we assumed that currently married women were sexually active, which was consistent with past studies [25,29,30,31,32,33]. The concept of unmet need for FP reflects the gap between the desire for childbearing and the use of FP methods. Using the Bradley et al. model [34], the unmet need for FP was computed. We categorized the unmet need for FP as “no unmet need or met need for FP (currently using FP),” “unmet need for spacing,” and “unmet need for limiting.”

### 2.4. Exposure Variables

For this study, the exposure variables were selected based on previous studies from LMICs [24,25,26,27,35] and availability in the DHS data. The study factors were broadly classified as community-level, predisposing, enabling, and need factors based on the Anderson conceptual model of health service utilization and health-seeking behaviors [36]. The adopted conceptual model was consistent with past studies that examined the relationship between study factors and the unmet need for FP among married women [24,25,26,35,37,38].

Community-level factors included the place of residence, which was categorized as ‘rural’ or ‘urban’. Predisposing included sociodemographic and health knowledge factors. Sociodemographic factors included women’s age (grouped as ‘15–24 years,’ ‘25–34 years,’ or ‘35–49 years’), parity (grouped as ‘zero,’ ‘one to four,’ or ‘five or more’), women’s and husband’s education (grouped as ‘no schooling’ or ‘primary education or higher’), women’s and husband’s employment (grouped as ‘no employment,’ ‘formal employment,’ or ‘informal employment’), household wealth index (grouped as ‘poor,’ ‘middle,’ or ‘rich’), and the number of partners a woman has (grouped as ‘one’ or ‘more than one partner’). Household wealth index was calculated by the National Bureau of Statistics and Inner-City Funds using principal component analysis (PCA), considering the ownership of household assets such as toilets, electricity, television, radio, fridge, and bicycle, as well as the availability of the source of drinking water and the floor material used for the main house [39].

**Figure 1:**
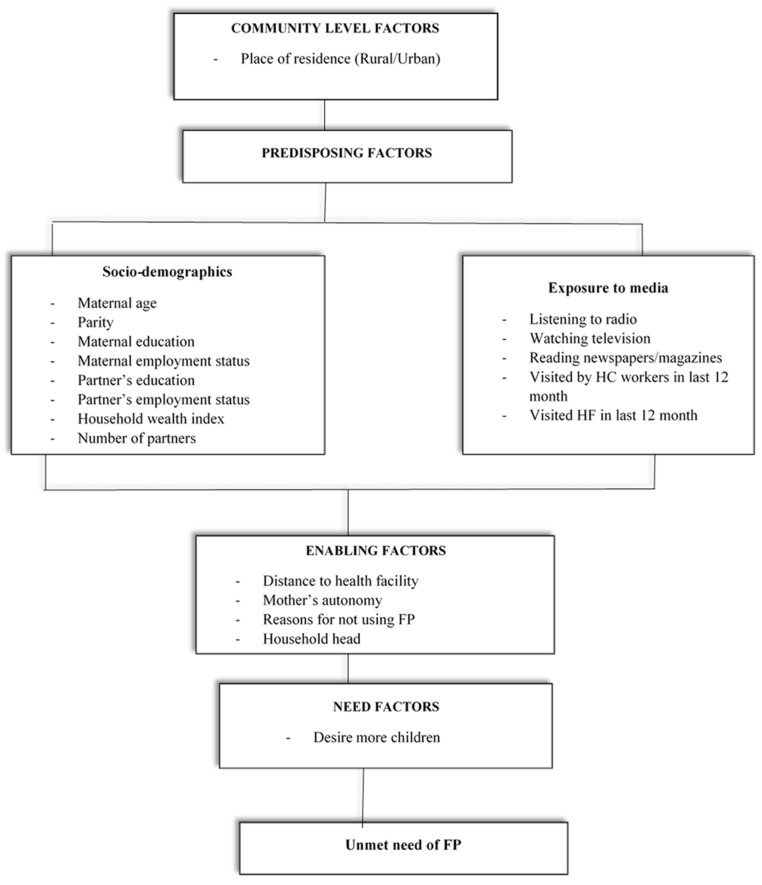
Conceptual model for unmet need for FP adopted from Anderson’s health service utilization model [36].

Health knowledge factors included listening to the radio, watching television, and reading newspapers/magazines (which were grouped as ‘yes’ or ‘no’), visit by health care workers within the last 12 months prior to the survey (grouped as ‘yes’ or ‘no’), and health facility visit within the last twelve months (grouped as ‘yes’ or ‘no’). Enabling factors included the distance to a health facility (grouped as ‘big problem’ or ‘not a big problem’ based on Measure DHS classification), women’s autonomy (grouped as ‘involved in household decision making’ or ‘not involved in household decision making’), household head (grouped as ‘male headed’ or ‘female headed’). The TDHS also collected information on reasons for not using FP methods, such as infrequent sex, sub-fecund, amenorrhea, breastfeeding, no need for more children, mother opposition, partner opposition, religion, not knowing FP methods, not knowing source of FP methods, fear of side effects, lack of access, too much cost, inconvenient to use FP methods, and interferes with body’s natural process (grouped as ‘yes’ or ‘no’). Need factors included planning to have more children (grouped as ‘do not want more,’ ‘want within two years,’ ‘want after two years and above or undecided’). All the study factors were grouped based on previous evidence from past studies from LMICs [24,25,26,27,31,38,40,41,42,43] (Figure 1).

### 2.5. Statistical Analysis

Our analytical approach was stepwise, based on previous studies conducted elsewhere [24,25,26,38,40,41,42,43]. The first step involved the estimation of frequencies and percentages of each study factor. Second, the prevalence of and trends in the unmet need for spacing and limiting births were calculated across the study factors (community-level, predisposing, enabling, and need factors) for each year from 1999 to 2016. Third, univariable logistic regression was conducted to establish associations between the study factors and the outcome variable of the unmet need for FP for spacing and limiting births. Fourth, multivariable logistic regression models were used to examine the associations between community-level, predisposing, enabling, and need factors with the outcome variable of the unmet need for FP. The multinomial regression model used in the analysis was based on the Anderson model [35] and was also based on other previous studies conducted in LMICs [24,25,26,31,35,37,38,40,41,42,43].

Community-level factor was entered into the stage 1 model to measure its association with the outcome variable, while adjusting for predisposing, enabling, and need factors. The same strategy was used for the predisposing factors (sociodemographic and health knowledge factors) model to establish the relationship with outcome variable, while adjusting for community-level, enabling, and need factors (stage 2). The corresponding modeling procedure was used for enabling factors and need factors in the third and fourth models (stage 3 and 4), respectively.

In addition, the multivariable regression model was conducted for combining the TDHS data from 1999 to 2016: (i) to examine the trends and factors associated with the unmet need for spacing and limiting; (ii) to provide the exceptional opportunity to compare the unmet need for FP over time; (iii) to improve the statistical power of the study.

Odds ratios (ORs) with 95% confidence intervals (CIs) were provided as the measure of association between the study variables and outcome variable. Analysis was performed using STATA version 14 software (Stata Corp, College Station, TX, USA), with the ‘svy’ command used to adjust for sampling weights, clustering, and stratification in both year-specific and combined datasets. The ‘mlogit’ function was used in the multinomial logistic regression models [44]. Furthermore, multi-collinearity was checked to examine for any influential variables related to each other using the ‘regress’ command [45] and reasons for not using FP were eliminated from the model due to high multi-collinearity.

## 3. Results

### 3.1. Characteristics of Study Participants

Over the study period from 1999 to 2016, 74% of the study participants resided in rural areas, and 38.4% were in the 25–34 years age group. Sixty percent of women had one to four children, and more than one-third (75.6%) of women attained a primary school education or higher. Informally employed women accounted for 70.8% of the study participants, and 51% of women resided in poor wealth status households. Eighty-eight percent of women resided in male-headed households, and 41.7% of women planned to have children after two years or more (Table 1).

### 3.2. Prevalence of Unmet Need for FP among Married Tanzanian Women

In the pooled data, the highest prevalence of the unmet need for FP for birth spacing was observed among women aged 15–24 years (21.3%), while the lowest prevalence was observed among women aged 35–49 years (3.6%) (Table 2). In the same data, the highest prevalence of the unmet need for FP for limiting births was found among women with five or more parity (18.1%), while the lowest prevalence was found among women aged 15–24 years (Table 3).

### 3.3. Trends in Unmet Need for FP in Tanzania, 1999–2016

**Figure 2:**
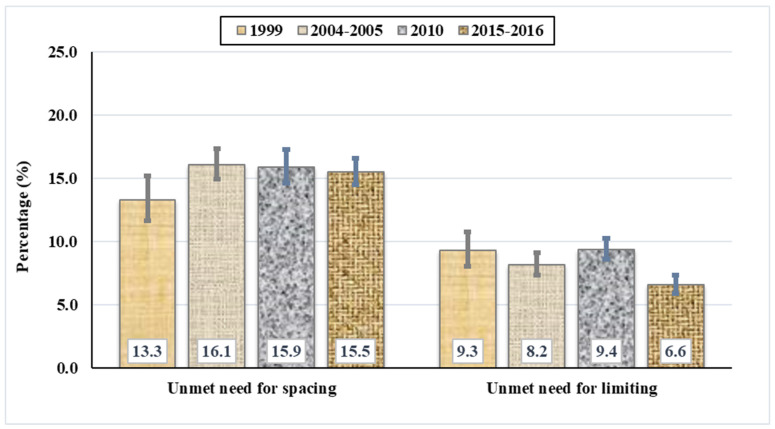
Trend in unmet need for family planning among married or in-union Tanzanian women, 1999 to 2016.

From 1999 to 2016, the unmet need for birth spacing among married women increased from 13.3% (95% confidence interval [CI]: 11.6%, 15.2%) in 1999 to 15.5% (95% CI: 14.5%, 16.6%) in 2016, with fluctuations between 2004/05 and 2010, with findings of 16.1% (95% CI: 14.9%, 17.3%) in 2004/05 and 15.9% (95% CI: 14.6%, 17.3%) in 2010. In contrast, the proportion of women with an unmet need for limiting births decreased from 9.3% (95% CI: 7.9%, 10.6%) in 1999 to 6.6% (95% CI: 5.9%, 7.3%) in 2016, with fluctuations in 2004/05 and 2010, with findings of 8.2% (95% CI: 7.5%, 9.1%) in 2004/05 and 9.4% (95% CI: 8.6%, 10.3%) in 2010 (Figure 2).

### 3.4. Factors Associated with Unmet Need for Spacing among Married Tanzanian Women

The unmet need for birth spacing was higher among women who resided in rural areas compared to those in urban areas (Odds Ratio [OR] = 1.41; 95% CI: 1.15, 1.71). Married women with one or more parity were more likely to have an unmet need for spacing compared to those with zero parity (OR = 4.67; 95% CI: 3.05, 7.17 for 1–4 parity and OR: 6.22; 95% CI: 3.94, 9.81 for five or more parity). Older women (25–34 and 35–49 years) had lower odds of having an unmet need for birth spacing compared to younger women (OR = 0.79; 95% CI: 0.68, 0.92 for 25–34 years and OR = 0.44; 95% CI: 0.34, 0.55 for 35–49 years) (Table 4).

Employed women had lower odds of having an unmet need for birth spacing compared to those who had no employment (OR = 0.60; 95% CI: 0.44, 0.84 for informal employment and OR = 0.68; 95% CI: 0.57, 0.81 for formal employment). The odds of having an unmet need for birth spacing was lower among women who watched television compared those who did not watch television (OR = 0.75; 95% CI: 0.63, 0.89) (Table 4).

### 3.5. Factors Associated with Unmet Need for Limiting among Married Tanzanian Women

Married women aged 25–34 years were associated with lower odds of having an unmet need for limiting births compared to those aged 15–24 years (OR = 0.64; 95% CI: 0.41, 0.98). Women with five or more parity were less likely to have an unmet need for limiting births compared to those with zero parity (OR = 6.06; 95% CI: 1.61, 22.84). Women who watched television had lower odds of having an unmet need for limiting births compared to those who did not watch television (OR = 0.76; 95% CI: 0.61, 0.96). Women who were not involved in household decisions were associated with lower odds of having an unmet need for limiting births compared to those who were involved in household decisions (OR = 0.64; 95% CI: 0.51, 0.81) (Table 5).

## 4. Discussion

We found that trends in the prevalence of the unmet need for limiting and spacing births varied between the years 1999 and 2016. Residing in rural areas, parity between 1–4 and 5+, and visiting a health facility for any health services within the last year were factors significantly associated with having an unmet need for FP for spacing of births. Women with parity of five and above were more likely to experience an unmet need for FP for limiting births. Women’s age between 25–34 and 35–49 years, women’s employment status (informal and formal), watching television, women’s autonomy, and planning to have more children were factors associated with lower odds of having an unmet need for FP for spacing of births. Women’s age between 25–34 years, watching television, women’s autonomy, and planning to have more children were factors associated with lower odds of having an unmet need for FP for limiting of births.

This study showed that employment status (both professional and non-professional workers) was associated with lower odds of having an unmet need for birth spacing compared to those who were not employed. This finding was supported by previous studies conducted in Ethiopia [46], India [47], and Ghana [38], respectively. The negative relationship between women’s occupation and the unmet need for birth spacing could be explained by improved household income and better autonomy for contraceptive decisions and use [38,46,47]. Therefore, the results of this study will inform policymakers and practitioners in developing interventions targeted at Tanzanian women to obtain gainful employment opportunities.

Previous studies conducted in Ghana, Malawi, and Zambia suggested that women’s occupation and an independent income positively improved a women’s autonomy by improving financial decision-making within the household, which can, in turn, lead to better contraceptive use decisions [38,48,49]. Additionally, professional and non-professional workers were observed to be significantly less likely to report an unmet need for birth spacing [50]. This study also found the same correlation between women’s autonomy and improved financial position with a reduced unmet need for FP. The likely reason for this association could be that employment status increases a woman’s autonomy in decision-making for health-seeking behavior, including FP, thus reducing the unmet need for FP [38].

Evidence shows that place of residence has a major impact on FP utilization and the unmet need for FP [51,52,53]. Our study found that women who resided in rural areas were more likely to have an unmet need for FP for birth spacing compared to women residing in urban settings. This finding was similar to studies conducted in Ethiopia [51,52,53], Burundi [53], Afghanistan [54], and Burkina Faso [24], in which living in rural areas was associated with the unmet need for FP. The possible reason as to why living in rural areas is associated with an unmet need for FP for birth spacing might be that women in rural areas are more likely to live far from health facilities, be less educated, and have less awareness of FP services compared to women residing in urban settings [51]. Our findings provide evidence that universal access to sexual and reproductive health-care services should be strengthened, including FP information and education, and reproductive health should be integrated into national strategies and programs through reinforcement of FP outreach in hard-to-reach areas and introduction of mHealth services [55]. There is also a need to continue investing financial resources in implementation of primary health care programs by establishing new health facilities close to rural areas, aligning with SDG-3, which aims to ensure healthy lives and promote wellbeing for all at all ages [56].

Parity was also found to be a significant predictor of the unmet need for FP (spacing and limiting of births). For birth spacing, parity between one and four and five and above was significantly associated with the unmet need for FP, while for limiting births, parity of five and above was associated with the unmet need for FP. As parity increased, the unmet need for FP increased, showing that having many children in Tanzania likely correlates with unplanned pregnancy for both FP spacing and limiting of births. This could be among the possible reasons as to why maternal mortality and morbidity remain high in the country. Our findings were consistent with other studies from LMICs such as Ethiopia [57,58], Sudan [29], Afghanistan [54], Nigeria [59], Rwanda [60], and Kenya [61]. This may be due to the fact that women with high parity may have high birthing experience, while another possible explanation might be that women who have given birth many times may have life experience with childbirth outcomes as well as the economical and health consequences of having many children on the mother [62]. Addressing the gap between the desired and actual number of children and contraceptive use remain key strategies for reducing the unmet need for FP among married women in Tanzania through information, education, and communication at all levels of the health system, especially in rural areas.

The study showed that visiting a health facility within the past twelve months prior to the surveys was significantly associated with an unmet need for FP. These findings were consistent with other studies conducted in LMICs, including Uganda, Senegal, Nepal [63], and Ethiopia [64,65]. This may indicate a lack of FP integration into maternal and/or other health services [51]. Visiting a health facility within 12 months for any health care reason and the unmet need for FP for birth spacing may be due to a lack of information and counseling aiming to improve FP service integration [53]. Our study findings provide support for FP integration at all levels within health facilities to all women who attend the health facility for any health reason, such as antenatal care (ANC), immunization of the baby, and any health-related issues in Tanzania.

Past studies conducted in LMICs such as Ghana [66], Zimbabwe [67], and India [68] suggested that women’s age was associated with a reduced experience of unmet need for FP. The current study showed a similar association between women’s age and decreased unmet need for FP for spacing and limiting of births, where women aged 25–34 and 35–49 years were associated with having an unmet need for birth spacing and women aged 25–34 years were associated with having an unmet need for limiting births. A possible reason for this association may be that younger women might not have reached the desired fertility age and hence prefer to space childbirth [69] rather than limit childbirth; however, our study observed the unmet need for limiting births among women aged 25–34 years, which was expected among older women who may have achieved the desired family size and no further pregnancies were desired [70,71,72]. Therefore, the study findings will inform policy makers in the formulation of policies targeting the unmet need for FP by addressing women according to their age-specific needs.

Our study showed that married/in-union women who were exposed to mass media (including watching television) had a decreased likelihood of having an unmet need for FP for both limiting and spacing births in Tanzania. The findings were consistent with other studies conducted in Burundi [53], Pakistan [73], Ethiopia [74], and Botswana [25], in which watching television was associated with a decreased unmet need for FP for both spacing and limiting births. The possible reasons for this association may impact FP use since television broadcasting may have provided appropriate health promotion messages, including FP use, its benefits, and the consequences of not using FP [25]. Based on Tanzanian economic reform, which involves rural electrification, many Tanzanians may be able to afford television [75], and the dissemination of maternal and newborn health promotion messages, including FP use, through television may contribute to a reduction in the unmet need for spacing and limiting births among Tanzanian married/in-union women.

Our findings indicated that women who were not involved in household decision-making had reduced odds of having an unmet need for FP for spacing and limiting births in Tanzania. This finding was supported by studies conducted in 32 Sub-Saharan African countries, which indicated that women who were not fully empowered were less likely to use modern FP [76]. Furthermore, studies conducted in Ghana [77], four Sub-Saharan African countries, namely Guinea, Mali, Namibia, and Zambia [78], and a study conducted in Namibia, Zambia, Uganda, and Ghana [79] showed that women’s participation in decision-making at the household level was associated with less FP use. The possible reasons for this association between women’s decision-making and decreased odds of having an unmet need for FP for spacing and limiting births may be that women who are not empowered to make household decisions are less likely to have high autonomy in health-seeking behavior, including maternal health services (FP use). The situation is likely to be worse if there are wide differences in socio-economic status between fathers and the presence of cultural norms that do not support women within their households and communities [80]. Tackling relevant sociocultural norms (such as acknowledging women’s worthiness, participation in decision-making, and abandoning norms that suppress women), bridging the economic and health gap, and eliminating gender inequalities in FP use are essential to increasing the health-seeking behavior of Tanzanian married women, including the use of FP services.

### 4.1. Implications on Practice and Policy

The study findings are expected to have a positive impact on policy and practice regarding reducing the unmet need for FP (limiting and spacing births). Policy makers are expected to formulate policies that will address the issues related to the unmet need for FP but also target all the attributes of unmet needs for FP. Doing so will change the practice of health professionals, thereby helping to reach many women with unmet needs for FP.

### 4.2. Limitations and Strengths

The study had the following limitations. First, the study was based on cross-sectional data, which makes it difficult to establish temporal relationships between the covariates and the unmet need for FP. However, the results of our study are consistent with other previous published longitudinal studies conducted in LMICs [33,38,81,82]. Second, the unmet need data collected in the past four TDHS were based on self-reported information and could be a source of recall bias. This may have caused misclassification bias in the outcome variable and can consequently lead to either over- or underestimation of the effect size. Third, the new definition of unmet need for FP and types of unmet need for spacing and limiting of births, incorporation of other biological issues that may impact fertility preferences, such as fecundity and postpartum amenorrhea, and unmet need for FP being limited to currently married women could result in a strong potential bias towards underestimating the true burden of the unmet need for FP particularly in populations were marriage is not a necessary precursor to sexual intercourse, assuming that all currently married women are sexually active. Fourth, there was a lack of assessment of all related confounders (such as data on access to health care services in terms of distance and women’s psychosocial factors) that would have provided further information regarding factors associated with the unmet need for FP in Tanzania.

However, the study had the following strengths. The larger sample size representing the general population and high response rates indicated that selection bias likely did not occur. The use of trained personnel and validated questionnaires in the TDHS also likely reduced measurement bias in the study. Finally, the study provides useful insight into key factors related to the unmet need for FP in Tanzania and potentially provides an opportunity for policymakers and public health practitioners to design and implement focused maternal health interventions aimed at improving FP uptake and hence reducing the unmet need for FP in Tanzania.

## 5. Conclusions

Our study shows that the unmet need for birth spacing increased during the study period among married Tanzanian women. However, the unmet need for limiting births decreased over the same study period. Women who resided in rural areas, with parity of one to four and five and above, had visited a health facility for any health services within the last twelve months, and planned to have more children after two years or were undecided when to have children had higher odds of experiencing an unmet need for birth spacing. The unmet need for limiting births was associated with women’s parity of five and above. Among married Tanzanian women, reducing the unmet need for FP, both for spacing and limiting births, is realistic if national and subnational health and social policies and programs focus on women who reside in rural areas and address all the amendable factors of parity, health facility use, and planning to have more children.

## Figures and Tables

**Table 1:** Characteristics of the study participants with an unmet need for FP in Tanzania, 1999–2016.

Variables	1999 (*N* = 2653)	2004/2005 (*N* = 6950)	2010 (*N* = 6412)	2015/2016 (*N* = 8210)	1999–2016 (*N* = 24,225)
	*n* (%)	*n* (%)	*n* (%)	*n* (%)	*n* (%)
**Community-level factors**					
Place of residence					
Urban	622 (23.4)	1647 (23.7)	1585 (24.7)	2535 (30.9)	6388 (26.4)
Rural	2031 (76.6)	5303 (76.3)	4827 (75.3)	5675 (69.1)	17,836 (73.6)
Sociodemographic factors					
Mother’s age					
15–24 years	777 (29.3)	1990 (28.6)	1610 (25.1)	2146 (26.1)	6523 (26.9)
25–34 years	1022 (38.5)	2803 (40.3)	2475 (38.6)	2994 (36.5)	9295 (38.4)
35–49 years	854 (32.2)	2157 (31.0)	2327 (36.3)	3070 (37.4)	8407 (34.7)
Parity					
Zero	225 (8.5)	551 (8.0)	377 (5.9)	592 (7.2)	1745 (7.2)
1–4	1530 (57.7)	4122 (59.3)	3893 (60.7)	5030 (61.2)	14,575 (60.2)
5+	897 (33.8)	2277 (32.8)	2141 (33.4)	2587 (31.5)	7903 (32.6)
Mother’s education					
No schooling	840 (31.7)	1994 (28.7)	1524 (23.8)	1559 (19.0)	5918 (24.4)
Primary education or higher	1813 (68.3)	4956 (71.3)	4887 (76.2)	6651 (81.0)	18,307 (75.6)
Mother’s employment status					
No employment	460 (17.4)	791 (11.4)	815 (12.7)	1657 (20.2)	3722 (15.4)
Formal employment	92 (3.5)	316 (4.6)	339 (5.3)	629 (7.7)	1376 (5.6)
Informal employment	2097 (79.2)	5842 (84.0)	5252 (82.0)	5924 (72.1)	19,115 (79.0)
Partner’s education					
No schooling	-	1291 (18.6)	998 (15.6)	1003 (12.2)	3292 (15.3)
Primary school	-	4991 (71.9)	4653 (72.7)	5612 (68.5)	15,256 (70.8)
Secondary education or higher	-	655 (9.5)	748 (11.7)	1584 (19.3)	2987 (13.9)
Partner’s employment status					
No employment	-	44 (0.6)	53 (0.8)	247 (3.0)	344 (1.6)
Formal employment	-	648 (9.3)	826 (12.9)	1030 (12.6)	2504 (11.6)
Informal employment	-	6255 (90.1)	5513 (86.3)	6933 (84.4)	18,701 (86.8)
Household wealth status					
Poor	1439 (57.8)	3987 (57.4)	1119 (46.7)	1441 (37.9)	7986 (51.1)
Middle	740 (29.7)	1873 (27.0)	815 (34.0)	1580 (41.6)	5008 (32.0)
Rich	312 (12.5)	1085 (15.6)	462 (19.3)	781 (20.5)	2640 (16.9)
Number of partners					
One	2008 (76.8)	5536 (79.6)	5146 (81.00)	6741 (82.1)	19,431 (80.4)
More than one	608 (23.2)	1415 (20.4)	1241 (19.0)	1466 (17.9)	4731 (19.6)
**Health knowledge factor**					
Listening to radio					
No	-	1708 (24.6)	1764 (27.5)	1918 (23.4)	5389 (25.0)
Yes	-	5238 (75.4)	4645 (72.5)	6292 (76.6)	16,275 (75.0)
Watch Television					
No	-	5403 (77.8)	4529 (70.7)	4436 (54.0)	14,368 (66.6)
Yes	-	1541 (22.2)	1882 (29.4)	3775 (46.0)	7198 (33.4)
Reading newspapers/Magazines					
No	-	4566 (65.7)	4298 (67.1)	5041 (61.4)	13,905 (64.5)
Yes	-	2379 (34.3)	2106 (32.9)	3168 (38.6)	7654 (35.5)
Visited by HC workers within last 12 month					
No	2463 (93.0)	6721 (96.7)	6100 (95.2)	7883 (96.0)	23,167 (95.7)
Yes	186 (7.0)	228 (3.3)	307 (4.8)	327 (4.0)	1048 (4.3)
Visited health facility for any health services within last 12 month					
No	947 (35.7)	2788 (40.1)	2093 (32.7)	2464 (30.0)	8292 (34.2)
Yes	1702 (64.3)	4162 (59.9)	4315 (67.3)	5742 (70.0)	15,921 (65.8)
**Enabling factors**					
Distance to health facilities					
Big problem	-	2775 (40.0)	5088 (79.5)	3692 (45.0)	11,553 (53.6)
Not a big problem	-	4170 (60.0)	1308 (20.5)	4519 (55.0)	9997 (46.4)
Mother’s autonomy					
Involved in all three household decisions	-	3306 (47.6)	3088 (48.2)	4671 (56.9)	11,065 (51.3)
Not involved in all three household decisions	-	3645 (52.4)	3324 (51.8)	3540 (43.1)	10,509 (48.7)
Household head					
Male	2320 (87.5)	6054 (87.1)	5700 (88.9)	7343 (89.4)	21,417 (88.4)
Female	333 (12.5)	896 (12.9)	711 (11.1)	868 (10.6)	2807 (11.6)
**Need factors**					
Future plan to have more children					
Want no more	865 (32.6)	2220 (32.0)	2055 (32.1)	2582 (31.5)	7722 (31.9)
Want within 2 years	744 (28.0)	1639 (23.6)	1365 (21.3)	1814 (22.1)	5561 (22.0)
Want after 2+ years	944 (35.6)	2903 (41.8)	2791 (43.6)	3468 (42.2)	10,106 (41.7)
Not sure	100 (3.8)	186 (2.7)	187 (3.0)	347 (4.2)	820 (3.4)

*n* (%): weighted count and proportion for each variable.

**Table 2:** Prevalence of unmet need for birth spacing.

Variables	1999 (*N* = 354)	2004/2005 (*N* = 1117)	2010 (*N* = 1021)	2015/2016 (*N* = 1275)	1999–2016 (*N* = 3767)	1999–2016
	*n* (%)	*n* (%)	*n* (%)	*n* (%)	*n* (%)	% Change (95% CI)
**Community-level factors**						
Place of residence						
Urban	81 (13.1)	190 (11.5)	179 (11.3)	335 (13.2)	784 (12.3)	0.2 (−3.9, 4.2)
Rural	272 (13.4)	928 (17.5)	843 (17.5)	941 (16.6)	2983 (16.7)	3.2 (0.8, 5.6)
Sociodemographic factors						
Mother’s age						
15–24 years	140 (18.1)	430 (21.6)	349 (21.7)	469 (21.9)	1389 (21.3)	3.8 (−0.2, 7.9)
25–34 years	172 (16.8)	530 (18.9)	479 (19.3)	565 (18.9)	1746 (18.8)	2.1 (−1.4, 5.5)
35–49 years	42 (4.9)	157 (7.3)	194 (8.3)	241 (7.8)	633 (7.5)	2.9 (0.8, 5.2)
Parity						
None	10 (4.5)	15 (2.7)	12 (3.2)	26 (4.4)	63 (3.6)	−0.1 (−3.7, 3.4)
1–4 children	258 (16.9)	807 (19.6)	700 (18.0)	887 (17.6)	2652 (18.2)	0.7 (−2.3, 3.8)
5+ children	85 (9.5)	295 (13.0)	310 (14.5)	363 (14.7)	1052 (13.3)	4.5 (1.9 (7.2)
Mother’s education						
No schooling	85 (10.2)	289 (14.5)	289 (18.9)	281 (18.0)	944 (16.0)	7.8 (3.8, 11.8)
Primary school and above	268 (14.8)	828 (16.7)	733 (15.0)	995 (15.0)	2824 (15.4)	0.2 9–2.1, 2.4)
Mother’s employment status						
No employment	88 (19.2)	124 (15.7)	174 (21.4)	313 (19.0)	699 (18.8)	−0.2 (−5.8, 5.3)
Formal employment	8 (8.3)	32 (10.0)	31 (9.0)	61 (9.7)	131 (9.5)	1.4 (−5.2, 7.9)
Informal employment	258 (12.3)	962 (16.5)	817 (15.6)	901 (15.2)	2937 (15.6)	2.9 (0.7, 5.1)
Partner’s education						
No schooling	-	168 (13.00	181 (18.1)	192 (19.2)	541 (16.4)	
Primary school	-	874 (17.5)	773 (16.6)	884 (15.8)	2531 (16.6)	
Secondary and higher	-	72 (10.9)	68 (9.0)	199 (12.5)	338 (11.3)	
Partner’s employment status						
No employment	-	5 (10.2)	3 (4.9)	37 (14.9)	44 (12.8)	
Formal employment	-	88 (13.5)	90 (10.9)	115 (11.2)	293 (11.7)	
Informal employment	-	1024 (16.4)	919 (16.7)	1123 (16.2)	3065 (16.4)	
Household wealth status						
Poor	192 (13.4)	664 (16.6)	236 (21.1)	296 (20.6)	1388 (17.4)	7.2 (3.7, 10.7)
Middle	94 (12.7)	320 (17.1)	134 (16.4)	191 (12.1)	738 (14.7)	−0.6 (−4.5, 3.3)
Rich	43 (13.8)	134 (12.3)	32 (6.9)	69 (8.9)	278 (10.5)	−4.9 (−10.8, 1.1)
Number of partners						
One	289 (14.4)	934 (16.9)	866 (16.8)	1098 (16.3)	3187 (16.4)	1.9 (−0.5, 4.2)
More than one	59 (9.7)	183 (13.0)	155 (14.6)	178 (12.1)	575 (12.1)	2.4 (−2.0, 6.8)
**Health knowledge factor**						
Listening to radio						
No	-	278 (16.3)	331 (18.8)	335 (17.5)	944 (17.5)	
Yes	-	838 (16.0)	690 (14.9)	940 (15.0)	2469 (15.3)	
Watch Television						
No	-	914 (16.9)	799 (17.7)	789 (17.8)	2502 (17.4)	
Yes	-	202 (13.1)	222 (11.8)	486 (12.9)	910 (12.6)	
Reading newspapers/Magazines						
No	-	743 (16.3)	735 (17.0)	855 (17.0)	2330 (16.8)	
Yes	-	374 (15.7)	289 (13.7)	421 (13.3)	1083 (14.2)	
Visited by HC workers within last 12 month						
No	324 (13.2)	1076 (16.0)	966 (15.8)	1246 (15.8)	3611 (15.6)	2.6 90.6, 4.8)
Yes	30 (16.0)	42 (18.2)	55 (17.9)	30 (9.0)	156 (14.9)	−7.0 (−16.2, 2.3)
Visited health facility within last 12 month						
No	89 (9.4)	300 (10.8)	243 (11.6)	289 (11.7)	922 (11.1)	2.3 (0.5, 5.1)
Yes	264 (15.5)	817 (19.6)	777 (18.0)	986 (17.2)	2844 (17.9)	1.7 (−0.9, 4.2)
**Enabling factors**						
Distance to health facilities						
Big problem	-	440 (15.9)	572 (15.3)	625 (16.9)	572 (15.3)	
Not a big problem	-	677 (16.2)	227 (16.8)	651 (14.4)	1292 (16.5)	
Mother’s autonomy						
Involved in all three household decisions	-	487 (14.7)	437 (14.2)	663 (14.2)	1587 (14.3)	
Not involved in all three household decisions	-	631 (17.3)	585 (17.6)	612 (17.3)	1827 (17.4)	
Household head						
Male	307 (13.2)	979 (16.2)	896 (15.7)	1134 (15.4)	3315 (15.5)	22.0 (−0.2, 45.9)
Female	46 (13.9)	139 (15.5)	126 (17.7)	141 (16.3)	452 (16.1)	23.7 (−4.9, 9.6)
**Need factors**						
Future plan to have more children						
Want no more	42 (4.9)	167 (7.5)	148 (7.2)	150 (5.8)	508 (6.6)	0.9 (−1.0, 3.0)
Want within 2 years	3 (0.4)	9 (0.5)	17 (1.2)	7 (0.4)	35 (0.6)	0.0 (−0.6, 0.6)
Want after 2+ years	278 (29.4)	874 930.1)	777 927.8)	1019 (29.4)	2947 (29.2)	0.0 (−4.5, 4.1)
Not sure	31 (30.7)	67 (36.1)	80 (43.0)	99 (28.5)	277 (33.8)	−2.2 (14.1, 9.7)

*n* (%); weighted count and proportional for each outcome variable by study factors reported in the 1999–2015/16 Tanzania Demographic and Health Survey with % point change indicating percentage point change from 1999 to 2015/16.

**Table 3:** Prevalence of unmet need for limiting births.

Variables	1999 (*N* = 246)	2004/2005 (*N* = 569)	2010 (*N* = 601)	2015/2016 (*N* = 540)	1999–2016 (*N* = 1956)	1999–2016
	*n* (%)	*n* (%)	*n* (%)	*n* (%)	*n* (%)	% Change (95% CI)
**Community-level factors**						
Place of residence						
Urban	45 (7.2)	129 (7.8)	131 (8.3)	166 (6.5)	470 (7.4)	−0.6 (−3.0, 1.7)
Rural	202 (9.9)	440 (8.3)	470 (9.7)	375 (6.6)	1486 (8.3)	−3.3 (−5.2, −1.5)
**Sociodemographic factors**						
Mother’s age						
15–24 years	27 (3.4)	35 (1.8)	16 (1.0)	20 (1.0)	98 (1.5)	−2.5 (−4.1, −0.8)
25–34 years	82 (8.0)	160 (5.7)	92 (3.7)	102 (3.4)	435 (4.7)	−4.6 (−6.9, −2.3)
35–49 years	138 (16.1)	375 (17.4)	493 (21.2)	418 (13.6)	1423 (16.9)	−2.5 (−6.3, 1.3)
Parity						
Zero	567 (0.3)	3 (0.5)	0 (0.0)	1 (0.0)	3 (0.2)	−0.2 (−0.7, 0.3)
1–4	92 (6.0)	134 (3.2)	138 (3.4)	158 (3.1)	522 (3.6)	−2.9 (−4.7, −1.1)
5+	153 (17.1)	433 (19.0)	463 (21.6)	381 (14.7)	1430 (18.1)	−2.3 (−5.9, 1.2)
Mother’s education						
No schooling	99 (11.8)	193 (9.7)	169 (11.1)	132 (8.4)	593 (10.0)	−3.4 (−6.4, −0.4)
Primary school and above	147 (8.1)	377 (7.6)	432 (8.9)	409 (6.1)	1363 (7.5)	−1.9 (−3.7, −0.1)
Mother’s employment status						
No employment	35 (7.6)	50 (6.3)	67 (8.3)	92 95.5)	244 (6.6)	−2.1 (−6.2, 2.0)
Formal employment	12 (12.9)	20 (6.3)	20 (6.0)	39 96.2)	91 (6.6)	−6.7 (−2.1, 7.3)
Informal employment	199 (9.5)	499 98.5)	512 (9.8)	410 96.9)	1620 (8.5)	−2.6 (−4.4, −0.8)
Partner’s education						
No schooling	-	120 (9.3)	122 (12.2)	67 (6.7)	309 (9.4)	
Primary school	-	409 (8.2)	427 (9.2)	388 (6.9)	1224 (8.0)	
Secondary and higher	-	39 (6.0)	52 (7.0)	85 (5.4)	176 (5.9)	
Partner’s employment status						
No employment	-	1 (2.3)	4 (7.1)	17 (6.9)	22 (6.3)	
Formal employment	-	44 (6.9)	88 (10.6)	54 95.3)	186 (7.4)	
Informal employment	-	524 (8.4)	509 (9.2)	469 96.8)	1502 (8.0)	
Household wealth status						
Poor	145 (10.1)	328 (8.2)	100 (8.9)	76 (5.3)	649 (8.1)	−4.8 (−7.3, −2.3)
Middle	66 (8.9)	173 (9.2)	77 (9.5)	89 (5.7)	405 (8.1)	−3.2 (−6.3, −0.1)
Rich	26 (8.3)	67 (6.2)	32 (6.8)	47 (6.0)	172 (6.5)	−2.3 (−7.3, 2.7)
Number of partners						
One	194 (9.6)	348 (7.9)	444 (8.6)	425 (6.3)	1500 (7.7)	−3.3 (−5.2, −1.9)
More than one	49 (8.0)	132 (9.3)	157 (25.8)	115 (7.9)	452 (9.4)	−0.2 (−3.0, 2.7)
**Health knowledge factor**						
Listening to radio						
No	-	174 (10.2)	220 (12.5)	149 (7.8)	543 (10.1)	
Yes	-	394 (7.5)	381 (8.2)	391 (6.2)	1166 (7.2)	
Watch Television						
No	-	477 (8.8)	479 (10.6)	331 (7.5)	1287 (9.0)	
Yes	-	91 (5.9)	122 (6.5)	209 (5.5)	422 (5.9)	
Reading newspapers/Magazines						
No	-	394 (8.6)	438 (10.2)	386 (7.7)	1217 (8.8)	
Yes	-	176 (7.4)	163 (7.8)	153 (4.8)	492 (6.4)	
Visited by HC workers within last 12 month						
No	230 (9.3)	540 (8.0)	577 (9.5)	519 (6.6)	1866 (8.1)	−2.8 (−4.4, −1.2)
Yes	16 (8.5)	29 (12.8)	24 (7.9)	21 (6.5)	90 (8.6)	−2.1 (−7.8, 3.6)
Visited health facility for any health services within last 12 month						
No	89 (9.4)	208 (7.5)	292 (13.9)	162 (6.6)	750 (9.1)	−2.8 (−5.7, −0.0)
Yes	157 (9.2)	361 (8.7)	309 (7.2)	379 (6.6)	1206 (7.6)	−2.6 (−4.6, −0.7)
**Enabling factors**						
Distance to health facilities						
Big problem	-	240 (8.6)	346 (9.3)	243 (6.6)	346 (9.3)	
Not a big problem	-	330 (7.9)	121 (8.9)	297 (6.6)	604 (7.7)	
Mother’s autonomy						
Involved in all three household decisions	-	299 (9.0)	318 (10.3)	360 (7.7)	977 (8.8)	
Not involved in all three household decisions	-	270 (7.4)	283 (8.5)	180 (5.1)	733 (7.0)	
Household head						
Male	203 (8.7)	472 (7.8)	529 (9.3)	463 (6.3)	1666 (7.8)	−2.4 (−4.0, −0.8)
Female	43 (13.1)	98 (10.9)	72 (10.2)	77 (8.9)	291 (10.4)	−4.2 (−9.4, 1.1)
**Need factors**						
Future plan to have more children						
Want no more	222 (25.7)	541 (24.4)	597 (29.0)	536 (20.8)	1896 (24.6)	−4.9 (−9.6, −0.2)
Want within 2 years	0 (0.0)	5 (0.3)	0 (0.0)	0 (0.0)	5 (0.008)	
Want after 2+ years	21 (2.2)	18 (0.6)	3 (0.1)	2 (0.06)	45 (0.4)	−2.2 (−3.5, −0.8)
Not sure	3 (2.0)	5 (2.7	1 (0.06)	2 (0.6)	11 (1.4)	−2.4 (−5.9, 1.0)

*n* (%); weighted count and proportional for each outcome variable by study factors reported in the 1999–2015/16 Tanzania Demographic and Health Survey with % point change indicating percentage point change from 1999 to 2015/16.

**Table 4:** Factors associated with unmet need for birth spacing in Tanzania, 1999 to 2016.

Variables	1999 (*N* = 354)	2004/2005 (*N* = 1117)	2010 (*N* = 1021)	2015/2016 (*N* = 1275)	1999–2016 (*N* = 3767)	*p* for Trend
	aOR (95% CI)	aOR (95% CI)	aOR (95% CI)	aOR (95% CI)	aOR (95% CI)	
**Community-level factors**						
Place of residence						
Urban	1.00	1.00	1.00	1.00	1.00	0.780
Rural	1.18 (0.64–2.02)	1.55 (1.17–2.05)	1.511 (0.96–2.37)	1.13 (0.83–1.55)	1.41 (1.15–1.71) **	0.145
**Sociodemographic factors**						
Mother’s age						
15–24 years	1.00	1.00	1.00	1.00	1.00	0.146
25–34 years	1.22 (0.79–1.88)	0.84 (0.68–1.04)	0.74 (0.52–1.05)	0.71 (0.51–0.99)	0.79 (0.68–0.92) **	0.864
35–49 years	0.68 (0.34–1.36)	0.41 (0.29–0.58)	0.42 (0.25–0.69)	0.54 (0.35–0.83)	0.44 (0.34–0.55) **	0.584
Parity						
Zero	1.00	1.00	1.00	1.00	1.00	0.298
1–4	2.50 (1.18–5.30)	5.52 (3.09–9.87)	13.95 (4.08–47.76)	2.87 (1.49–5.54)	4.67 (3.05–7.17) **	0.535
5+	2.26 (0.95–5.41)	7.18 (3.89–13.25)	20.42 (5.48–76.01)	3.98 (1.92–8.24)	6.22 (3.94–9.81) **	0.236
Mother’s education						
No schooling	0.00	1.00	1.00	1.00	1.00	0.267
Primary school and above	0.98 (0.64–1.50)	0.93 (0.75–1.16)	1.02 (0.69–1.50)	1.01 (0.68–1.48)	0.98 (0.83–1.16) **	0.476
Mother’s employment status						
No employment	1.00	1.00	1.00	1.00	1.00	0.987
Formal employment	0.47 (0.22–1.01)	0.92 (0.49–1.70)	0.51 (0.26–0.97)	0.54 (0.34–0.85)	0.60 (0.44–0.84) **	0.124
Informal employment	0.66 (0.44–0.99)	0.85 (0.64–1.11)	0.43 (0.29–0.64)	0.79 (0.59–1.07)	0.68 (0.57–0.81) **	0.133
Partner’s education *						
No schooling	-	1.00	1.00	1.00	1.00	0.004
Primary school	-	1.25 (0.94–1.66)	1.00 (0.69–1.45)	0.89 (0.58–1.40)	1.12 (0.91–1.38)	0.910
Secondary and higher	-	0.85 (0.53–1.35)	0.80 (0.42–1.54)	0.73 (0.43–1.03)	0.85 (0.63–1.14)	0.316
Partner’s employment status *						
No employment	-	1.00	1.00	1.00	1.00	0.873
Formal employment	-	0.89 (0.34–2.38)	1.42 (0.34–5.99)	1.03 (0.53–1.99)	1.07 (0.61–1.88)	0.003
Informal employment	-	0.87 (0.34–2.18)	1.94 (0.50–7.44)	1.32 (0.68–2.56)	1.27 (0.73–2.22)	0.024
Household wealth status *						
Poor	1.00	1.00	1.00	1.00	1.00	0.000
Middle	0.84 (0.55–1.28)	1.11 (0.90–1.37)	0.81 (0.57–1.16)	0.87 (0.63–1.21)	0.98 (0.84–1.15)	0.068
Rich	0.91 (90.47–0.73)	1.04 (0.76–1.43)	0.42 (0.21–0.81)	0.99 (0.59–1.69)	0.89 (0.69–1.16)	0.262
Number of partners						
One	1.00	1.00	1.00	1.00	1.00	0.268
More than one	0.99 (0.59–1.64)	1.14 (0.92–1.39)	1.43 (0.95–2.17)	1.04 (0.74–1.45)	1.17 (0.99–1.38)	0.324
**Health knowledge factor**						
Listening to radio *						
No	-	1.00	1.00	1.00	1.00	0.012
Yes	-	1.01 (0.82–1.24)	1.22 (0.81–1.84)	0.93 (0.68–1.28)	1.00 (0.85–1.18)	0.936
Watch Television *						
No	-	1.00	1.00	1.00	1.00	0.029
Yes	-	0.74 (0.58–0.96)	0.95 (0.62–1.47)	0.69 (0.52–0.94)	0.75 (0.63–0.89) **	0.443
Reading newspapers/Magazines *						
No	-	1.00	1.00	1.00	1.00	0.131
Yes	-	1.12 (0.92–1.37)	0.95 (0.65–1.39)	0.92 (0.68–1.25)	1.01 (0.87–1.18)	0.656
Visited by HC workers within last 12 month						
No	1.00	1.00	1.00	1.00	1.00	0.102
Yes	1.13 (0.49–2.58)	0.97 (0.62–1.51)	1.16 (0.63–2.15)	0.28 (0.12–0.67)	0.77 (0.55–1.07)	0.027
Visited health facility for any health services within last 12 month						
No	1.00	1.00	1.00	1.00	1.00	0.038
Yes	1.23 (0.86–1.75)	1.38 (1.14–1.67)	1.11 (0.77–1.60)	1.09 (0.81–1.47)	1.25 (1.07–1.45) **	0.983
**Enabling factors**						
Distance to health facilities *						
Big problem	-	1.00	1.00	1.00	1.00	0.012
Not a big problem	-	1.11 (0.93–1.33)	1.21 (0.84–1.76)	0.89 (0.65–1.22)	1.06 (0.92–1.24)	0.713
Mother’s autonomy *						
Involved in all three household decisions	-	1.00	1.00	1.00	1.00	0.525
Not involved in all three household decisions	-	1.17 (0.99–1.38)	0.59 (0.36–0.96)	1.07 (0.85–1.34)	0.80 (0.65–0.99)	0.093
Household head						
Male	1.00	1.00	1.00	1.00	1.00	0.579
Female	1.49 (0.80–2.77)	1.13 (0.86–1.49)	0.94 (0.63–1.41)	1.31 (0.85–2.01)	1.12 (0.91–1.38)	0.218
**Need factors**						
Plan to have more children						
Want no more	1.00	1.00	1.00	1.00	1.00	0.471
Want within 2 years	0.38 (0.01–0.29)	0.49 (0.02–0.10)	0.06 (0.02–0.23)	0.12 (0.04–0.39)	0.063 (0.04–0.11) **	0.747
Want after 2+ years	5.26 (3.07–9.03)	2.94 (2.28–3.79)	2.44 (1.60–3.73)	8.31 (4.62–14.97)	3.54 (2.89–4.32) **	0.090
Not sure	5.85 (2.91–11.79)	5.16 (3.07–8.68)	7.11 (2.99–16.92)	8.42 (4.91–14.44)	5.54 (3.95–7.79) **	0.966

aOR: adjusted Odds Ratio; * variable not reported in the 1999 Tanzania Demographic and Health Survey. In the model of community-level factors, adjustments were conducted for predisposing (sociodemographic and media exposure), enabling, and need factors. Similar approaches were used for the predisposing, enabling, and need factors with adjustments for respective factors in multivariate models; ** variables are statistically significant.

**Table 5:** Factors associated with unmet need for limiting births in Tanzania, 1999 to 2016.

Variables	1999 (*N* = 246)	2004/2005 (*N* = 569)	2010 (*N* = 601)	2015/2016 (*N* = 540)	1999–2016 (*N* = 1956)	*p* for Trend
	aOR (95% CI)	aOR (95% CI)	aOR (95% CI)	aOR (95% CI)	aOR (95% CI)	
**Community-level factors**						
Place of residence						
Urban	1.00	1.00	1.00	1.00	1.00	0.242
Rural	1.51 (0.81–2.80)	0.99 (0.67–1.48)	0.91 90.47–1.77)	0.77 (0.49–1.22)	0.99 (0.76–1.29)	0.086
Sociodemographic factors						
Mother’s age						
15–24 years	1.00	1.00	1.00	1.00	1.00	0.257
25–34 years	1.12 (0.49–2.58)	0.74 (0.43–1.25)	0.63 (0.21–1.87)	0.38 (0.13–1.08)	0.64 (0.41–0.98) **	0.002
35–49 years	1.08 (0.39–2.96)	0.69 (0.41–1.18)	0.91 (0.30–2.80)	0.57 (0.21–1.51)	0.69 (0.45–1.06)	0.259
Parity						
Zero	1.00	1.00	1.00	1.00	1.00	0.542
1–4	6.08 (0.78–47.42)	2.17 (0.54–8.76)	3.95 (2.07–7.79)	16.61 (1.04–265.19)	3.32 (0.89–12.38)	0.450
5+	6.05 (0.74–49.67)	4.96 (1.18–20.80)	11.06 (5.66–26.11)	20.99 (1.35–326.32)	6.06 (1.61–22.84) **	0.584
Mother’s education						
No schooling	1.00	1.00	1.00	1.00	1.00	0.556
Primary school and above	0.84 (0.54–1.29)	0.85 (0.64–1.13)	0.90 (0.51–1.60)	0.99 (0.58–1.69)	0.88 (0.70–1.11)	0.058
Mother’s employment status						
No employment	1.00	1.00	1.00	1.00	1.00	0.002
Formal employment	0.87 (0.19–3.83)	0.59 (0.26–1.39)	0.41 (0.16–1.06)	1.29 (0.65–2.54)	0.74 (0.48–1.15)	0.093
Informal employment	0.66 (0.30–1.44)	0.73 (0.47–1.15)	0.79 (0.43–1.44)	0.77 (0.48–1.23)	0.79 (0.60–1.05)	0.531
Partner’s education*						
No schooling	-	1.00	1.00	1.00	1.00	0.359
Primary school	-	1.01 (0.73–1.39)	0.52 (0.29–0.94)	0.64 (0.35–1.19)	0.83 (0.64–1.06)	0.026
Secondary and higher	-	0.89 (0.51–1.58)	0.66 (0.27–1.61)	0.96 (0.43–2.14)	0.95 (0.64–1.40)	0.102
Partner’s employment status *						
No employment	-	1.00	1.00	1.00	1.00	0.397
Formal employment	-	5.56 (1.75–17.61)	2.32 (0.32–16.57)	0.69 (0.25–1.91)	1.68 (0.82–3.45)	0.007
Informal employment	-	4.89 (1.62–14.73)	1.42 (0.20–9.98)	1.08 (0.41–2.83)	1.67 (0.83–3.35)	0.263
Household wealth status						
Poor	1.00	1.00	1.00	1.00	1.00	0.922
Middle	0.85 (0.53–1.37)	1.24 (0.89–1.70)	0.90 (0.54–1.51)	0.75 (0.47–1.20)	1.08 (0.85–1.37)	0.013
Rich	1.08 (0.47–2.47)	0.92 (0.58–1.45)	0.42 (0.15–1.22)	0.57 (0.29–1.09)	0.81 (0.57–1.16)	0.063
Number of partners						
One	1.00	1.00	1.00	1.00	1.00	0.904
More than one	0.74 (0.45–1.21)	0.94 (0.69–1.28)	1.52 (0.92–2.53)	1.24 (0.79–1.94)	1.07 (0.86–1.34)	0.587
**Health knowledge factor**						
Listening to radio *						
No	-	1.00	1.00	1.00	1.00	0.063
Yes	-	0.74 (0.55–0.99)	0.55 (0.31–0.97)	1.59 (0.94–2.70)	0.82 (0.65–1.04)	0.277
Watch Television *						
No	-	1.00	1.00	1.00	1.00	0.266
Yes	-	0.74 (0.52–1.05)	1.05 (0.59–1.87)	0.97 (0.59–1.56)	0.76 (0.61–0.96) **	0.087
Reading newspapers/Magazines *						
No	-	1.00	1.00	1.00	1.00	0.405
Yes	-	1.09 (0.82–1.44)	1.18 (0.70–2.00)	0.78 (0.52–1.16)	1.02 (0.83–1.26)	0.013
Visited by HC workers within last 12 month						
No	1.00	1.00	1.00	1.00	1.00	0.132
Yes	0.74 (0.45–1.21)	1.38 (0.84–2.28)	1.70 (0.72–4.03)	0.97 (0.45–2.08)	1.30 (0.90–1.88)	0.053
Visited health facility for any health services within last 12 month						
No	1.00	1.00	1.00	1.00	1.00	0.706
Yes	0.89 (0.37–2.18)	1.49 (1.18–1.90)	0.75 (0.49–1.14)	1.04 (0.63–1.72)	1.16 (0.96–1.42)	0.008
**Enabling factors**						
Distance to health facilities *						
Big problem	-	1.00	1.00	1.00	1.00	0.170
Not a big problem	-	1.02 (0.79–1.32)	1.39 (0.81–2.40)	0.95 (0.59–1.51)	1.07 (0.87–1.31)	0.145
Mother’s autonomy *						
Involved in all three household decisions	-	1.00	1.00	1.00	1.00	0.121
Not involved in all three household decisions	-	1.09 (0.88–1.35)	0.67 (0.34–1.34)	1.12 (0.72–1.75)	0.64 (0.51–0.81) **	0.180
Household head						
Male	1.00	1.00	1.00	1.00	1.00	0.517
Female	1.39 (0.75–2.58)	1.35 (0.98–1.87)	0.80 (0.41–1.55)	1.21 (0.64–2.32)	1.08 (0.83–1.41)	0.259
**Need factors**						
Plan to have more children						
Want no more	1.00	1.00	1.00	1.00	1.00	0.297
Want within 2 years	2.52 (1.72–3.70)	0.01 (0.00–0.06)	7.41 (4.96–11.10)	1.47 (0.99–2.20)	0.01 (0.00–0.03) **	0.995
Want after 2+ years	0.10 (0.04–0.22)	0.03 (0.02–0.05)	0.01 (0.00–0.03)	0.00 (0.00–0.03)	0.02 (0.01–0.03) **	0.001
Not sure	0.12 (0.03–0.42)	0.14 (0.05–0.39	5.31 (2.42–11.72)	0.03 (0.00–0.20)	0.08 (0.03–0.19) **	0.075

aOR: adjusted Odds Ratio; * variable not reported in the 1999 Tanzania Demographic and Health Survey. In the model of community-level factors, adjustments were conducted for predisposing (sociodemographic and media exposure), enabling, and need factors. Similar approaches were used for the predisposing, enabling, and need factors with adjustments for respective factors in multivariate models; ** variables are statistically significant.

## Data Availability

The data used in this article are available to download for researchpurposes upon registration; https://www.dhsprogram.com/data/available-datasets.cfm.
